# Higher-order chromatin domains link eQTLs with the expression of far-away genes

**DOI:** 10.1093/nar/gkt857

**Published:** 2013-10-01

**Authors:** Geet Duggal, Hao Wang, Carl Kingsford

**Affiliations:** Lane Center for Computational Biology, Carnegie Mellon University, 5000 Forbes Avenue Pittsburgh, PA, USA

## Abstract

Distal expression quantitative trait loci (distal eQTLs) are genetic mutations that affect the expression of genes genomically far away. However, the mechanisms that cause a distal eQTL to modulate gene expression are not yet clear. Recent high-resolution chromosome conformation capture experiments along with a growing database of eQTLs provide an opportunity to understand the spatial mechanisms influencing distal eQTL associations on a genome-wide scale. We test the hypothesis that spatial proximity contributes to eQTL-gene regulation in the context of the higher-order domain structure of chromatin as determined from recent Hi-C chromosome conformation experiments. This analysis suggests that the large-scale topology of chromatin is coupled with eQTL associations by providing evidence that eQTLs are in general spatially close to their target genes, occur often around topological domain boundaries and preferentially associate with genes across domains. We also find that within-domain eQTLs that overlap with regulatory elements such as promoters and enhancers are spatially more close than the overall set of within-domain eQTLs, suggesting that spatial proximity derived from the domain structure in chromatin plays an important role in the regulation of gene expression.

## INTRODUCTION

Expression quantitative trait loci (eQTL) experiments map mutations in a genome to variation in gene expression (1). They have led to the discovery of regulators driving the expression of genes (2), genes associated with disease point mutations [single nucleotide polymporphisms (SNPs)] and molecular targets for cancer therapy (3). Determining theraputic targets and identifying regulators for disease genes hinges on our ability to determine the mechanisms by which a mutation modulates the expression of a gene. We ask here whether the higher-order 3D structure of chromatin plays a role in determining eQTL associations on a genome-wide scale by placing eQTLs in close spatial proximity to their genomically distant genes. Anecdotal evidence suggests that spatial proximity contributes to the regulation of genes for specific eQTLs (4–7). However, the extent of this phenomenon is unknown. Recent genome-wide analyses suggest that the topological structure of chromatin may be associated with eQTL associations. For example, SNPs from genome-wide association studies were observed to be depleted in DNA fragments of RNA polymerase-mediated chromatin interaction networks (8). This polymerase-specific approach considers SNPs from genome-wide association studies, but not eQTLs and their target genes. eQTLs have also been analyzed with respect to how predictive a variety of chromatin features are for the target genes associated with eQTLs (9) as well as in the context of other chromatin markers such as DNaseI hypersensitive sites, transcription factor binding sites and promotor regions (10). Some of these features such as DNaseI hypersensitivity imply the topological structure of chromatin may be related to eQTL associations, but the issue of whether higher-order properties of chromatin structure are linked with eQTL-gene associations on the whole remains open. It is now possible to compare eQTL associations with chromatin structure at a genome-wide scale using data from higher-coverage Hi-C experiments (11) [a type of chromosome conformation capture; see (12,13) for reviews], which afford the observation of chromatin interactions at resolutions as high as 20 kb.

**Figure 1.**
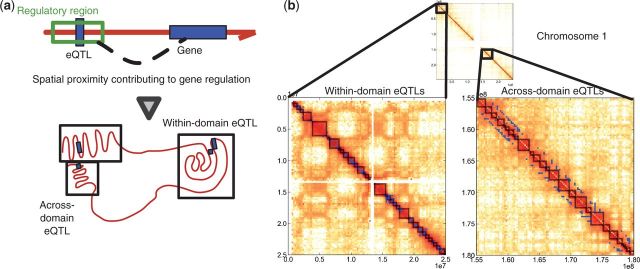
Spatial proximity of eQTLs and their target genes in the context of the higher-order domain structure of chromatin. (**a**) Schematic for how mutations in regulatory regions can affect the expression of spatially close target genes. Closer-range higher-frequency interactions could contribute to within-domain gene regulation and longer-range cross-domain interactions could transiently bring two domains in close spatial proximity to contribute to ultra-long range regulation. (**b**) Experimental evidence suggesting both inter- and intra-domain regulation of gene expression from SNP mutations. Two sections of a heat map of chromatin interactions for chromosome 1 fibroblast interactions derived from the Hi-C experiments of Dixon *et al.* (top) show that both intra-domain interactions (bottom left) and inter-domain interactions (bottom-right) are prevalent. Each axis represents a locus on chromosome 1. Higher-frequency interactions between a pair of loci are red. Interactions between eQTLs and genes from a recent database (eQTL browser, eqtl.uchicago.edu) are shown in blue, and domains are enclosed in black squares along the diagonal of the matrix.

We determine whether spatial proximity plays a role in eQTLs regulating their target genes as illustrated in Figure 1 by placing them in the context of genomically contiguous spatially compact domains that have been shown to be persistent across cell types and conserved across species (11). These domains are highly correlated with a number of chromatin markers associated with gene regulation and may therefore be associated with the positions of eQTLs on the genome. Specifically, we test the following hypotheses: (i) eQTL fragments interact often with other fragments; (ii) eQTLs are genomically close to domain boundaries; (iii) eQTLs are spatially close to their target genes, especially within domains; (iv) eQTLs often associate with genes across domains; and (v) within-domain eQTLs with regulatory elements are close to their target genes. We argue that the higher-order structure of chromatin is coupled with eQTL associations by providing evidence for each of these hypotheses.

For our analysis, we gathered 112 302 eQTL–gene pairs from a database of eQTLs (eQTL Browser, eqtl.uchicago.edu) spanning 10 publications and six cell types (L. Mangravite *et al.* submitted for publication) (10,14–21). Of these, we selected the 89 103 intrachromosomal pairs (nearly 80% of all pairs) that have a SNP at least 50 kb from the boundary of their associated genes. These eQTL–gene pairs were then mapped onto Hi-C interaction networks for individual chromosomes using data from Dixon *et al.* (11) at the resolution of 40 kb (see ‘Chromatin interaction networks and topological domains’ of ‘Materials and Methods’ section). As Hi-C data have an inherent resolution limit, we aggregated eQTL–gene pairs that cannot be distinguished with respect to their Hi-C interactions by grouping pairs that correspond to the same Hi-C fragments into a collection of 15 661 eQTL–gene equivalence classes (see ‘eQTL equivalence classes and properties’ of ‘Materials and Methods’ section). To perform statistical tests, we used an optimal matching framework that associates eQTLs and domains with randomly generated counterparts while controlling for a variety of confounding variables including genomic properties of eQTLs, interaction patterns common in 3C experiments and topological domain lengths (see ‘Optimal matching framework’ and ‘Statistical analysis’ of ‘Materials and Methods’ sections). This conservative approach minimizes the chance that conclusions drawn from our analysis are artifacts of less interesting aspects of data derived from eQTL and Hi-C experiments. Despite taking such a controlled approach, the significant relationship we observed between eQTLs, their target genes and chromatin structure suggests a clear genome-wide link between chromatin structure and the landscape of eQTL associations.

## MATERIALS AND METHODS

### Chromatin interaction networks and topological domains

We constructed chromatin interaction networks for various cell types using the genome-wide Hi-C assays from Dixon *et al.* (11) (embryonic stem cells, IMR90 fibroblasts). We built networks for chromosomes 1 through 22 individually because within-chromosome interactions can be analyzed at higher resolutions than across-chromosome interactions (22).

For each chromosome, Hi-C data in its raw form can be represented as a weighted graph 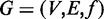 where vertices in *V* are restriction fragments and the frequency of interaction *f* (*e*) observed for an edge 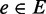 is the number of observed Hi-C read pairs that map to the associated restriction fragments. Owing to the limited resolution of Hi-C experiments, we constructed a graph 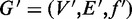 that binned interactions at a genomic resolution of 40 kb. Specifically, the vertices in 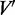 partition a chromosome into 40 kb fragments (intervals on the chromosome). The frequency 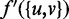 of an interaction 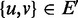 is the sum of interaction frequencies for all interactions in *E* with read pairs mapping within the fragments *u* and *v*. The total frequency of a fragment *v* is then defined as 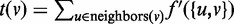. The higher-coverage experiments in Dixon *et al.* afford the use of an error-correction method by Yaffe and Tanay (23), and we used the normalized data Dixon *et al.* provide at the 40 kb resolution (combined data set, http://chromosome.sdsc.edu/mouse/hi-c/download.html).

We obtained topological domains for each chromosome from Dixon *et al.* Domain boundaries for the Hi-C assays described earlier in the text were identified using a hidden Markov model (Supplementary Information in Dixon *et al.*) and published in the same location as the Hi-C data. Each domain in this sequence of topological domains 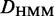 is an interval 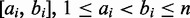 such that no pair of distinct domains overlap, and *n* is the number of fragments in 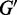 for a given chromosome. To further verify our conclusions, we also perform our statistical tests on a recent alternative definition of domains (24) that have been shown to be different than those of Dixon *et al.*, yet still enriched for similar chromatin marks. The domain-finding method of Dixon *et al.* uses a Hidden Markov Model with a local ‘directionality index’ statistic, whereas the method for the alternative definition explicitly encodes the quality score of a domain in a dynamic program. Dixon *et al.*’s method results in domains at a particular scale of genomic length (in this case, ∼1 megabase), whereas the alternative definition identifies domains that persist across multiple length scales.

### eQTL equivalence classes and properties

eQTLs were obtained from the eQTL browser (eqtl.uchicago.edu), which compiles genome-wide eQTL data from 10 publications [cortex (17), fibroblast (14), liver (18,21), lymphoblast (L. Mangravite *et al.* submitted for publication) (10,14,15,19,20), monocyte (16) and T-cell (14) cell types]. We intersected gene names in this database with gene names or IDs in the Ensembl database (25) and select genes that are associated with a unique range in the Ensembl database to produce a collection *Q* of eQTLs.

We represent an eQTL–gene pair 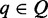 as a SNP paired with the gene with which it is associated. It is possible that a single gene can be correlated with different SNPs, all of which lie on the same Hi-C fragment (we call this a SNP or eQTL fragment). These eQTL associations are indistinguishable in the chromatin interaction network and would all share the same interaction frequencies. We therefore defined equivalence classes 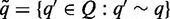 where 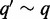 if the SNPs in *q* and 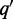 map to the same vertex in the chromatin interaction network 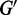 and the target genes are the same. We then constructed a new set of eQTL–gene pairs as defined by the subsets of equivalent eQTL-gene pairs 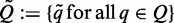. Each such eQTL–gene equivalence class 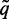 is then represented by a SNP fragment, a gene name and the set of fragments associated with the gene.

Recall that *t*(*v*) is the total frequency of edges incident on *v* in 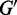. We define five properties for an eQTL–gene pair 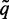:

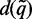
 is the **SNP-gene distance**: the genomic distance between the midpoint of SNP fragment and the midpoint position of the closest gene fragment to the SNP.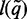 is the **gene length**, the genomic distance between the midpoints of the first and last fragment of the gene.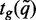, the **maximum total gene frequency**, is the maximum *t*(*v*) of any fragment 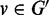 overlapping the gene in this eQTL. This represents the extent to which the gene associated with this eQTL interacts with other fragments in the Hi-C graph.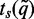, the **total SNP frequency**, is *t*(*s*) where *s* is the SNP fragment in 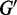 associated with 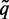. This is the extent to which the SNP associated with this eQTL interacts with other fragments in the Hi-C graph.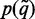 is the **spatial proximity**: the maximum frequency 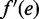 of all interactions 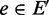 between the SNP fragment and fragments overlapping the gene. This is a measure of how close the SNP and gene are based on interactions in the Hi-C graph.

### Optimal matching framework

To test for statistical significance while controlling for confounding variables, we compared the set of eQTL–gene pairs 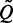 and the sequence of topological domains *D* to their randomly generated counterparts. To control for confounding variables associated with the genomic properties of eQTL–gene pairs, interaction patterns prevalent in 3C experiments and properties associated with topological domains, we matched the eQTLs in 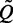 with randomly defined eQTLs 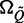 and the sequence of topological domains *D* with a set of randomly defined domain sequences 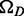 while keeping the confounding variables between the matched elements as similar as possible.

**Figure 2.**
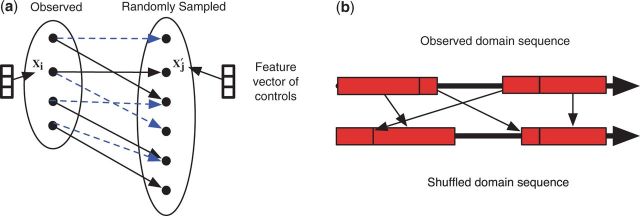
Optimal matching framework and domain shuffling procedure. (**a**) Observed elements (eQTLs or a sequence of domains) are paired with their two (k = 2) best-matched random counterparts: black arrows are the best match, and blue dotted arrows are the second-best match. Each observed and random element is associated with a feature vector of controlled variables, and small Euclidean distances between these vectors correspond to better matches. (**b**) An illustration of a domain shuffling where domains are red and non-domains are gaps along a chromosome. The shuffling is performed so that it preserves domain and non-domain lengths as well as the observed sequence of domains and non-domains.

To do this, we associate each observed element *i* in 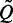 or *D* with a numerical feature vector of confounding variables 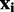 and each element *j* in the sample spaces 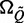 or 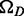 with a feature vector of the same counfounding variables 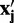. Features for **x** are defined differently for eQTLs and domains (see ‘Statistical analysis of eQTLs' and ‘Statistical analysis of topological domains' of ‘Materials and Methods' section). Using a maximum weight bipartite matching solver (26), each observed element *i* is matched to *k* random elements *j* such that for all matched pairs 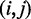, the sum of Euclidean distances 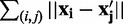 is minimized (Figure 2a).

### Sample space for spatial proximity

For a particular chromosome, we designed the sample space 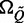 to be considerably larger than the set of eQTLs 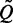 so that close matches between the real and randomly generated eQTLs are more likely. Specifically, we created random eQTL equivalence classes and added them to 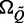 via this procedure: Let *n_p_* be a large number that defines how many times larger the randomly generated set of eQTLs is in comparison with the real set of eQTLs. While 
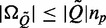
:

1. To construct a randomized gene, we select a random gene length *l* from the sequence of gene lengths observed for this chromosome.
2. Select a random SNP–gene distance *d* from the sequence of distances observed for this chromosome.
3. Randomly decide whether the SNP occurs upstream or downstream of the gene based on the observed probability of a SNP occurring upstream or downstream of a gene.
4. If the SNP occurs downstream, select a random SNP location *s* in the interval 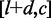 where *c* is the chromosome length. Otherwise, set *s* to be a random location in the interval 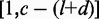. Build a random eQTL 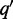 from these properties and determine its equivalence class 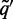.
5. Add 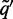 to 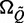.

In practice, we observe that the target size 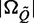 is always reached within 10 million iterations. To ensure that a maximum weight bipartite matching solver does not match an eQTL to itself, we match to elements in 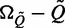. We set 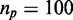 for all our tests.

### Statistical analysis of eQTLs

To determine whether a set of eQTLs has significantly higher spatial proximity than expected by chance, each feature vector for an equivalent eQTL–gene pair 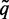 contains four features described earlier in the text: 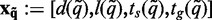. To calculate whether eQTLs in 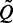 have greater spatial proximity after an optimal matching (*k* = 1) with their counterparts in 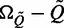, we use the non-parametric Wilcoxon signed-rank test on 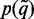 for matched pairs, a standard procedure in matched case–control studies (27).

### Sample space for topological domains

A sample space for topological domains allows us to explicitly determine whether a test statistic on the observed set of domains and eQTLs differs significantly from random alternatives for domains. An alternative approach could be to use the sample space for eQTLs defined earlier to test whether a test statistic on the observed set of domains and eQTLs differs significantly from the same statistic on random eQTLs. However, in the instances when we use the sample space for topological domains, we are primarily interested in whether the observed domain structure is significant as opposed to the observed set of eQTLs. For a particular chromosome, an element of the sample space for topological domains was generated by randomly shuffling domain and non-domain regions from 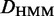 on the chromosome, preserving the domain and non-domain lengths as well as the observed pattern of domains and non-domains in the process (Figure 2b). The following procedure selects from two shuffled lists of domain and non-domain lengths so that both properties are preserved:

1. Label all intervals on the chromosome associated with a topological domain in 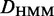 as ‘domain’. Label all intervals not overlapping with a domain interval as ‘non-domain’.
2. Generate a pair for each interval *i*: (‘domain’, *l_i_*) or (‘non-domain’, *l_i_*) where *l_i_* is the length of interval *i*. Let *O* be a list of these pairs that represents the observed ordering of domain and non-domain intervals on the chromosome.
3. Create two lists from *O*: one with just domains 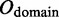 and another with just non-domains: 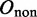.
4. Randomly shuffle both 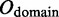 and 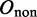.
5. Generate new domain positions by traversing the observed sequence *O* of domains and non-domains. If a domain is encountered in *O*, remove the last domain region from 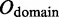 and append it to a list of region lengths *L*. Otherwise, remove the last non-domain region from 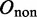 and append it to *L*.
6. For each domain in *L*, assign it a start position on the chromosome based on the sum of region lengths occurring before the domain in *L*.

The set of *n_d_* shuffled domain sequences is then defined as 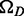. We set *n*_d_ = 10 000 for all our tests.

### Statistical analysis of topological domains

To determine whether eQTL–gene pairs cross domains more often than expected by chance, we calculated the probability of observing a number of domain crossings *n* or higher for a sequence of domains. Specifically, we define the ‘SNP–gene interval’ as the range between the midpoint of the SNP fragment for an eQTL–gene pair and the midpoint of the closest fragment of its target gene. If the SNP–gene interval of an eQTL–gene pair overlaps, but is not completely contained within, a domain, it is said to ‘cross’ the domain. The distance of this interval for an eQTL 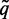 is 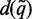.

We calculate this probability while controlling for two random variables:

- *N_G_*: the number of genes in eQTL–gene pairs crossing a random sequence of domains, and
- *N_S_*: the number of SNP fragments in eQTL–gene pairs crossing a random sequence of domains.

The probability or *P*-value that *n* or more eQTL–gene pairs cross domains is then conditioned on the number of genes and SNPs crossing domains being within a range of the observed number of genes and SNP fragments crossing domains:

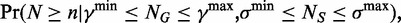

where *N* is a random variable representing the number of eQTL–gene pairs crossing a random sequence of domains. The parameters γ and σ, allow the properties of a random sequence of domains to be similar, but not exactly equal to, the observed sequence of domains 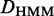. We first created a set 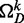 to determine the ranges for γ and σ and then used this set to determine the sample space of topological domains 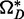. Specifically, we determined the values of the parameters by comparing a sequence of domains *D* to its 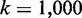 best matches in the sampled space: 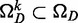. The feature vector used for matching sampled domains to observed domains 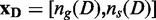 contains the number of genes in eQTL–gene pairs crossing domains and the number of SNP-fragments in eQTL–gene pairs crossing domains.

We set the parameter range for γ to 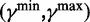 based on ranges in 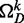 according to:

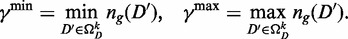

A similar procedure is performed for σ using 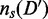 instead of 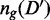. The conditioned sample space 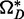 for the *P*-value is then defined as a subset of the sample space 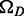:

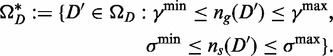



This procedure of determining the top- *k* matches in the control set and restricting the parameters to ranges observed in this size- *k* set guarantees that there will be at least *k* samples in 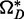 while otherwise letting parameter ranges be as close to the observed domain sequence as possible. When compared with no restrictions on the parameters, we observe that this approach is typically much more conservative in the *P*-values it obtains, as it is less likely that statistical significance is obtained from confounding variables. We always include the observed domain sequence in 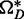 to guarantee *P* > 0.

## RESULTS

All results reported are for the IMR90 fibroblasts from the Dixon *et al.* Hi-C experiment (11). We define ‘across-domain’ eQTL–gene pairs as those that have SNP–gene intervals that overlap but are not completely contained within a domain as defined earlier in the text (see ‘Statistical analysis' of ‘Materials and Methods' section). eQTLs that do not associate with genes across a domain are said to be ‘non-crossing’. Non-crossing eQTLs can be further subdivided into those that associate within-domain and those in gapped regions between domains. We determined that of the 6283 non-crossing eQTL pairs, 6068 (97%) occur within domains. Therefore, the results presented for non-crossing pairs generally apply to within-domain as well.

### eQTLs interact often

**Figure 3.**
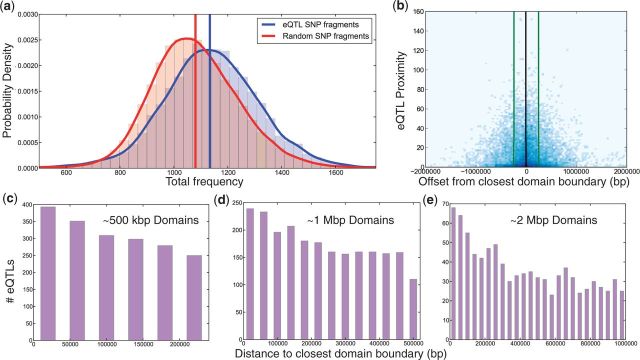
Occurrence and spatial proximities of eQTL fragments and associated genes near domain boundaries. (**a**) Distribution of the total number of interactions (total frequency) for all Hi-C fragments (red) versus eQTL fragments (blue). The vertical red and blue lines represent the means of each distribution, and the distribution outlines were obtained from kernel density estimates of each distribution. (**b**) The 2D histogram representing the relationship between eQTL spatial proximity and the genomic offset to the closest domain boundary. Vertical green lines represent −250 and 250 kb offsets from the boundary. (**c, d, e**) Histograms of the genomic distance between a SNP fragment and its closest domain boundary for SNP fragments occurring within 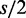 bp of a domain boundary. s is the size of the domain. Domain sizes plotted are 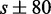 kb, where s = 500 kb, 1 Mb and 2 Mb.

We found that eQTL SNP fragments *v* have higher total Hi-C interaction frequencies *t*(*v*), on average, than the background set of all Hi-C fragments (Figure 3a, 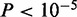, Wilcoxon rank sum test) and that fragments containing domain boundaries also have higher total Hi-C interaction frequencies when compared with the background set of Hi-C fragments (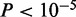, Wilcoxon rank sum test). Additionally, we found that fragments containing both eQTLs and domain boundaries exhibited higher average total interaction frequencies than when considering the set of all eQTL fragments (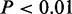, Wilcoxon rank sum test). Together, these three results suggest that fragments containing eQTLs and domain boundaries tend to interact often with other regions of the chromosome. Fragments with eQTLs may therefore be spatially close to other fragments in the genome, and we test specifically whether eQTLs are spatially close to their target genes in a subsequent section.

### eQTLs are genomically close to domain boundaries

We next asked whether eQTL fragments are genomically close to domain boundaries. Even though eQTLs can occur as far as 3.94 Mb away from a domain start point, we observed that 6757 of 10 899, nearly 62%, of the eQTL fragments lie within just 250 kb of a domain boundary (*P* < 0.001 for randomly shuffled domains; Figure 3b, vertical green lines). Figure 3b also indicates that the spatial proximity 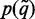 of an eQTL–gene pair is larger near the boundaries. We also found that the preferential occurrence of eQTLs around domain boundaries is not limited to domains of small or large length. Figure 3c–e illustrates that the distribution of distances to the closest domain boundary are skewed toward 0 for three different domain lengths (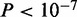, Bonferroni-corrected chi-squared test for all lengths).

### eQTLs are spatially close to their target genes

We determined whether eQTL fragments from eQTL–gene pairs are spatially proximate to their associated gene fragments using the definition of spatial proximity discussed earlier in the text. As eQTLs can affect genes of different length across different distances, and fragments involved in eQTL–gene pairs can vary significantly in their total frequencies, we paired each eQTL–gene association with its best-matched randomly defined counterpart, where a good match is defined as a small Euclidean distance between two feature vectors: one associated with the eQTL and the other with the random counterpart (see ‘Optimal matching framework' of ‘Materials and Methods' section). The feature vector contained the properties of the equivalent eQTL: SNP–gene distance, gene length, total SNP fragment frequency and maximum total gene fragment frequency (see ‘eQTL equivalence classes and properties’ of ‘Materials and Methods' section). By comparing each eQTL with its best-matched random counterpart in this way, we conservatively controlled for confounding variables, which, if ignored, could result in less meaningful, and perhaps artifactual interpretations of eQTL–gene spatial proximity.

**Figure 4.**
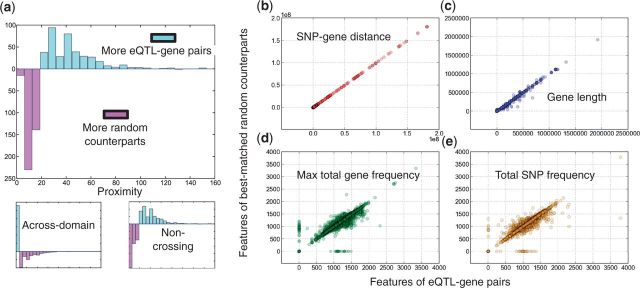
Spatial proximity of eQTL–gene pairs when compared with their optimally matched randomly defined counterparts. (**a**) Spatial proximity bins (*x*-axis) for which we observe more equivalent eQTLs (cyan) or more randomly selected eQTL–gene pairs (magenta). The bottom two plots are the same as the top except restricted to observed eQTLs that are exclusively across-domain or non-crossing. (**b–e**) Correlation plots for real eQTL control features on the *x*-axis and their random counterparts on the *y*-axis. Features controlled for are (b) SNP–gene genomic distance, (c) gene length, (d) maximum total gene 3C interaction frequency and (e) total SNP 3C interaction frequency.

We found that eQTL–gene pairs with spatial proximity 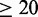 (‘high proximity’) occurred more often in the set of observed eQTLs than in the set of randomly defined, optimally matched counterparts (Figure 4a). Pairs with spatial proximity 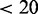 occured more frequently in the set of random eQTLs. In general, the set of matched pairs differed significantly (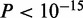, Wilcoxon signed rank test for matched pairs) from one another with respect to spatial proximity. Figures 4b–e verify that individual dimensions of the feature vectors are matched well across all eQTLs with Pearson correlation coefficients of ρ = 0.99, 0.99, 0.96, 0.95 for SNP–gene distance, gene length, total SNP fragment frequency and maximum total gene frequency respectively. The correlations are very high, but not 1, as the best match is based on the Euclidean distance between feature vectors, which does not require that matches are simultaneously perfect in every dimension. In practice, we find that eQTLs match well with their random counterparts in many dimensions simultaneously.

We also determined that high-proximity non-crossing eQTL–gene pairs occur more often in the set of observed eQTLs. The opposite was true for across-domain pairs (Figure 4a, bottom) for which high proximity pairs occurred at a lower frequency than the matched pairs. The overall spatial proximity of eQTLs is therefore largely due to non-crossing eQTL associations. Together, these analyses suggest that interactions between eQTLs and their distant, within-domain genes, are generally enriched for higher-frequency Hi-C interactions even after controlling for a variety of confounding factors.

### eQTLs often associate with genes across domains

**Figure 5.**
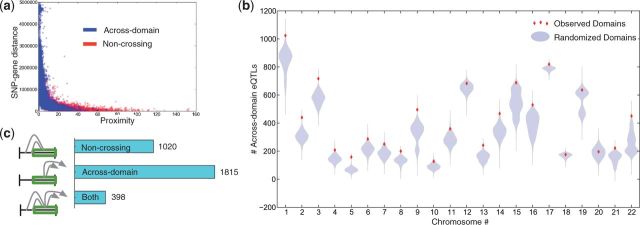
Spatial proximity of eQTLs acting across domains versus not. (**a**) Scatterplot of across-domain (blue) and non-crossing (red) eQTL interactions for SNP–gene distance versus spatial proximity. (**b**) Distribution of across-domain eQTL counts for randomly shuffled domains (violin plots) versus the set of observed domains (red diamonds) for chromosomes 1–22. (**c**) Number of eQTL SNP fragments with at least two distinct target genes interacting exclusively across domains, exclusively not, or both.

We also investigated the extent to which eQTL–gene pairs interact across domain boundaries versus those that do not cross domain boundaries. For all eQTL–gene pairs, we found that 9378 interact across domains, whereas 6283 are non-crossing. Although across-domain eQTL–gene pairs tend to be lower-proximity and interact over longer distances than those that do not, pairs of relatively large proximity still interact across domains with 1074 such pairs with spatial proximities between 10 and 30 (Figure 5a). To test the significance of the number of eQTLs crossing domain boundaries, we generated random sequences of domains controlling for a variety of properties associated with the observed sequence of domains: domain and non-domain lengths, the sequence of domains and non-domains, the number of SNP fragments crossing domains and number of genes crossing domains (see ‘Sample space for topological domains' and ‘Statistical analysis' of ‘Materials and Methods' section). We compared the number of eQTL–gene pairs crossing real domains to the corresponding number of eQTL–gene pairs crossing random domains and found that eQTL associations across domain boundaries occur more often than expected by chance (Benjamini–Hochberg-corrected 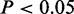 for 17/22 chromosomes, Figure 5b). We also observed that SNP fragments interacting with more than one gene typically do so either entirely across domains or entirely within a domain—only 398 of 3233, 12%, of all multi-gene SNP fragments involve both crossing and non-crossing associations (Figure 5c).

### Within-domain eQTLs with regulatory elements are close to their genes

**Figure 6.**
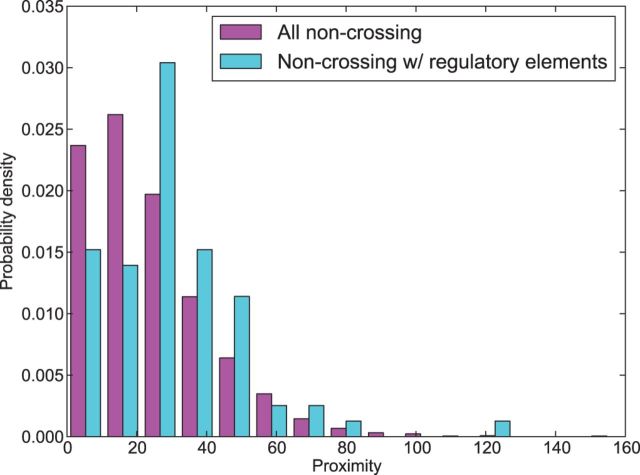
Distribution of non-crossing eQTLs with regulatory elements (cyan) versus the set of all non-crossing eQTLs (magenta).

We tested the hypothesis that distal within-domain eQTL interactions involving regulatory elements as compiled by Wang *et al.* (9) are spatially more proximate than the set of all eQTLs interacting within domain. Of the 364 eQTLs associated with these regulatory elements, 130 associate with genes at least 50 kb away from the SNP, forming 97 equivalence classes. Of these, 74 are non-crossing (i.e. mostly within domains) and are associated with a considerably larger proximities than the remaining set of non-crossing eQTLs (Figure 6; 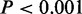, Wilcoxon rank sum test).

## DISCUSSION

Our analysis maps eQTL–gene associations onto the 3D domain structure of chromatin using the 3C graph and provides compelling evidence that spatial proximity is a relevant mechanistic component for how a mutation affects the expression of its correlated gene, especially via regulatory regions within domains. We identified a number of confounding variables that could lead to artifactual conclusions when relating eQTLs to the spatial structure of chromatin and controlled for them using an optimal matching resampling-based framework. This approach is conservative not only because it directly controls for confounding variables but also because it does not rely on an analytical definition of a null model that, under such a controlled setting, would be difficult to justify and could easily result in statistical significance where a resampling scheme would not (28).

We found that non-crossing eQTL associations account for almost all of the enrichment in spatial proximity we observe when compared with their best-matched random counterparts (Figure 4a). This suggests that these pairs are likely to be spatially close and that those interacting across domains may do so via more transient interactions between tightly coupled chromatin domains. In line with this picture, our observation that most eQTLs interact exclusively across or not across domains may be closely linked to the tightly packed nature of chromatin domains. If an eQTL interacts in a more tightly packed region of chromatin, it is more likely to be inaccessible to regions farther away in the genome than an eQTL in a more accessible region near the domain boundary. This is consistent with the observation that domain boundaries exhibit an increased accessibility as measured by DNaseI sensitivity (11). The fact that domain boundaries are, by design, enriched for Hi-C interactions (11) partially explains our observation that eQTL fragments have generally higher frequency, as eQTLs tend to fall near domains, but it is not clear why eQTLs occur so often around domain boundaries. Finally, the prevalence of across-domain eQTLs suggests that the variation in gene expression observed in eQTLs may be driven largely by mutations in open regions of chromatin near domain boundaries.

We also verified that our analysis is robust to different Hi-C interaction matrices, domain sequences and subsets of eQTLs. When using Hi-C data for the embryonic stem cell type instead of the IMR90 fibroblast, we still observed largely similar patterns. However, the spatial proximity distributions as shown in Figure 3a were less separated, yet still significantly different from one another. The set of eQTLs we test is an aggregation from many recent experiments, but we also found that running the analysis on individual experiments, especially those with more eQTLs (>50 000) (L. Mangravite *et al.* submitted for publication) (16), resulted in similar conclusions as the overall set of eQTLs. The study by Schadt *et al.* (18) with ∼5000 eQTL associations resulted in similar conclusions when compared with the set of all eQTLs. Although other smaller studies also resulted in similar enrichments, they occasionally did not reproduce the domain-size-specific boundary plots in Figure 3c–e. In those studies, fewer chromosomes were enriched for eQTLs associating across domains as shown in Figure 5b. We observed that an alternative set of domains resulting from a recently developed procedure (24) resulted in similar conclusions, and that the statistic for the number of eQTLs crossing domains was far more significant than when using the domains identified by Dixon *et al.* Together, these analyses suggest that the properties we observe are not artifacts of a particular set of eQTLs, Hi-C interaction matrix or sequence of domains.

The spatial proximity of eQTLs within and across domains may also underpin the correlations of eQTLs with other features of chromatin as determined from recent analyses (9,29). Supporting this, a recent analysis by Wang *et al.* (9) found that Hi-C data can be used to predict regulatory elements and their target promoters. We showed that eQTLs near regulatory regions identified by Wang *et al.* are enriched for spatial proximity to their targets: i.e. they are closer to their within-domain targets than the set of all eQTLs that occur within domain. eQTLs could also be near regulatory regions that have not yet been identified, and this test therefore establishes that the enrichment for spatial proximity is specific to the regulatory regions (and transcription factors used to identify these regions) defined by Wang *et al.* SNPs from eQTL associations have also been shown to be highly correlated with SNPs from recently identified DNaseI sensitivity QTLs (30), which are mutations correlated with variation in chromatin accessibility. DNaseI sensitivity QTLs may therefore be associated with chromatin domains in a similar way as we have shown eQTLs to be.

Our observations correlating topological domains with eQTL positions suggest a new hypothesis: disrupting the observed domain structure near an eQTL could result in a significant change in expression of its target gene. For example, if an eQTL associated with an enhancer and its target gene occur within a topological domain (e.g. an eQTL from the set of within-domain eQTLs associated with regulatory elements analyzed earlier), an experimental technique that could separate them so they are no longer spatially close would allow this hypothesis to be tested. A recent publication suggests a technique to split or merge topological domains (31), which could conceivably be applied to test this hypothesis.

The geometry of chromatin structure has provided numerous insights into the regulation of gene expression, nuclear organization and cancer (13,32–35). Our results provide strong evidence that chromatin structure is coupled with the placement of eQTLs on the genome. The techniques used in our analysis can be directly applied to other types of genomic data where pairs of interacting elements are involved (e.g. enhancer-promoter pairs) and can easily be extended to control for additional confounding variables. As more tissue-specific higher-resolution chromatin interaction networks are constructed, our framework may help to more specifically understand how chromatin structure differentially influences pairwise associations across cell types.

## FUNDING

National Science Foundation (NSF) [CCF-1256087, CCF-1053918 and EF-0849899]; National Institutes of Health (NIH) [1R21AI085376 and 1R21HG006913]; Alfred P. Sloan Research Fellow (to C.K.). Funding for open access charge: NIH [IR21HG006913].

*Conflict of interest statement*. None declared.
